# Transradial access with intra-aortic catheter looping for the treatment of intracranial aneurysms

**DOI:** 10.3389/fneur.2023.1128960

**Published:** 2023-04-27

**Authors:** Gang-Qin Xu, Yue-Yang Ba, Dong-Yang Cai, Bo-Wen Yang, Tong-Yuan Zhao, Jiang-Yu Xue, Tian-Xiao Li, Bu-Lang Gao

**Affiliations:** Endovascular Treatment Center, Henan Provincial People's Hospital, Zhengzhou University, Zhengzhou, China

**Keywords:** intracranial aneurysms, catheter looping, endovascular treatment, transradial access, intra-aortic looping

## Abstract

**Purpose:**

The study aimed to investigate the feasibility and effect of transradial access with intra-aortic catheter looping for the treatment of intracranial aneurysms.

**Materials and methods:**

This retrospective one-center study was performed on patients with intracranial aneurysms which were embolized through transradial access with intra-aortic catheter looping because of the difficulty of transfemoral access or transradial access without intra-aortic catheter looping. The imaging and clinical data were analyzed.

**Results:**

A total of 11 patients were enrolled, including seven (63.6%) male patients. Most patients were associated with one or two risk factors of atherosclerosis. There were nine aneurysms in the left internal carotid artery system and two aneurysms in the right internal carotid artery system. All 11 patients had complications with different anatomic variations or vascular diseases, which made endovascular operation via the transfemoral artery difficult or a failure. The right transradial artery approach was adopted in all patients, and the success rate of intra-aortic catheter looping was 100%. Embolization of intracranial aneurysms was successfully completed in all patients. No instability of the guide catheter was encountered. No puncture site complications or surgical-related neurological complications occurred.

**Conclusion:**

Transradial access with intra-aortic catheter looping for embolization of intracranial aneurysms is technically feasible, safe, and efficient as an important supplementary approach to the routine transfemoral access or transradial access without intra-aortic catheter looping.

## Introduction

Compared with the transfemoral artery approach, the transradial artery approach has become the preferred method for diagnosis and intervention of coronary heart diseases because of the advantages of lower bleeding complications at the puncture site, more comfortability, and a shorter hospital stay (1–3). In recent years, neurointerventional physicians have paid increasingly more attention to transradial artery access, and transradial access has been widely used for cerebral angiography (4–6). Moreover, the use of transradial access for embolizing intracranial aneurysms is also on the rise (7–10). The key to successful transradial embolization of cerebral aneurysms is to navigate the guiding catheter to the right location to obtain good support before the endovascular embolization of the aneurysm. Nonetheless, for patients with a small arterial angle between the subclavian (or innominate) artery and the common carotid artery similar to that in the case of the bovine arch (the right and left common carotid arteries stem from the same trunk), a lower vertebral artery opening, type III aortic arch (all supra-aortic vessels originate below a straight line with an acute angle between the vessel origin and the aortic arch) (11), or a need for superselection of the contralateral vertebral artery, it is often very difficult to navigate the guiding catheter to the right location using the conventional approaches or to stably maintain the guiding catheter for successful embolization after the guiding catheter has been sent to the right position (12, 13). In these cases, looping of the guiding catheter within the aortic lumen (intra-aortic catheter looping) through the transradial artery access may be helpful and has been successfully applied in cerebral angiography and cerebrovascular stenting (14–19). Nonetheless, no studies have been conducted until now to investigate the effect and feasibility of transradial intra-aortic catheter looping for the treatment of intracranial aneurysms. It was hypothesized that this approach was feasible and efficient in the abovementioned difficult situations in endovascular treatment of intracranial aneurysms. This retrospective study was consequently performed to explore the effect and feasibility of transradial intra-aortic catheter looping for the treatment of intracranial aneurysms.

## Materials and methods

This retrospective one-center cohort study was approved by the ethics committee of our hospital, and all patients or their family members had signed the informed consent to participate. Between January 2018 and November 2022, patients with intracranial aneurysms who had a small arterial angle formed between the subclavian (or innominate) artery and the common carotid artery similar to that in the bovine arch, a lower vertebral artery opening, type III aortic arch, a need for superselection of the contralateral vertebral artery, and failure of transfemoral access for endovascular treatment of intracranial aneurysms were retrospectively enrolled to experience the transradial intra-aortic catheter looping for embolizing the intracranial aneurysms (Table 1). Patients with intracranial aneurysms who did not have the above difficult situations or who did not undergo the transradial intra-aortic catheter looping technique were excluded. Patients with aortic valve insufficiency, valvular vegetation, or ascending aortic disease were also excluded (Table 1).

**Table 1:** Inclusion and exclusion criteria.

**Inclusion criteria**	**Exclusion criteria**
Bovine arch	Without the use of the transradial intra-aortic catheter looping technique
A lower vertebral artery opening	Aortic valve insufficiency
Type III aortic arch	Aortic valvular vegetation
A need for superselction of the contralateral vertebral artery	Ascending aortic disease
Failure of transfemoral access	

### Endovascular procedure and intra-aortic catheter looping

All patients underwent the modified Allen test (20) before surgery to evaluate collateral circulation, and the test results were negative.

The endovascular treatment was performed under general anesthesia. A 6F radial sheath was routinely inserted into the right radial artery after a puncture of the radial artery, and 5,000 units of heparin and 200 micrograms of nitroglycerin were administered through the sheath. A SIM2 catheter (Cordis, Miami Lakes, FL, USA) was used for cerebral angiography to show cerebral aneurysms. With the guidance of a 0.035-inch 150 cm loach guide wire (Vietnam, TERUMO, Japan), a 0.70 Navien 115/125 cm intermediate catheter (Medtronic, Irvine, CA, USA) was navigated to the ascending aorta through the right subclavian artery, and the loach guide wire was looped within the ascending aorta. Thereafter, the intermediate catheter was slowly looped by slowly following the loach guide wire and navigated into the target parent artery. A V18 guide wire (COSTARICA, Boston Scientific, MA, USA) was sent through the intermediate catheter to the C2 segment of the right internal carotid artery before withdrawing the loach guidewire, and the V18 guide wire remained to support and prevent the intermediate catheter from folding at the loop position. After the intermediate catheter was sent as the guiding catheter to the right location, a working angle was selected before introducing a microcatheter whose tip had been appropriately shaped into the aneurysm cavity for coiling. In cases of wide-necked aneurysms, stent-assisted coiling was performed using an intraluminal support device LVIS Jr stent (Microvention, COSTARICA, France) which was half deployed before dense coiling.

### Perioperative medication

Patients who needed stent implantation at preoperative evaluation were given dual antiplatelet therapy composing aspirin 100 mg/day and clopidogrel 75 mg/day 5 days before the endovascular procedure. After embolization, the dual antiplatelet therapy was continued for half a year before switching to aspirin 100 mg/day for the long term. Patients with a ruptured intracranial aneurysm were slowly given tirofiban 8.0 μG/kg through intravenous injection followed by tirofiban application at the dose of 0.10 μ G/kg/min for maintenance, and 300 mg aspirin and 300 mg clopidogrel were administered after embolization. After 2 h, tirofiban was stopped, and aspirin 100 mg/day and clopidogrel 75 mg/day were continued for half a year before switching to aspirin 100 mg/d for long-term application (21).

### Statistical analysis

The SPSS software (version 19.0, IBM, Chicago, IL, USA) was used for statistical analysis. Categorical data were presented as frequency and percentages. The statistical significance was set at a *p* <0.05.

## Results

A total of 11 patients were enrolled (Table 2), including seven (63.6%) male patients. Most patients were associated with one or two risk factors of atherosclerosis (Table 2). There were nine aneurysms in the left internal carotid artery system and two aneurysms in the right internal carotid artery system (Table 3). All 11 patients had complications with various anatomic variations or vascular diseases, which made endovascular operation via the femoral artery difficult or a failure.

**Table 2:** Baseline data of patients (frequency and percentage).

**Variables**	**Data**
Age (y)	53–78 (mean 56)
Sex (M)	7 (63.6%)
Hypertension	10 (90.9%)
Diabetes mellitus	6 (54.5%)
Hyperlipidemia	9 (81.8%)
Smoking	7 (63.6%)
Femoral/iliac artery occlusion	2 (18.2%)
Severe tortuosity of abdominal aorta	3 (27.3%)
Type III aortic arch	5 (45.5%)
Scoliosis	1 (9.1%)

**Table 3:** Angiographic data of patients.

Patients with left posterior communicating artery aneurysm	4 (36.4%)
**Patients with left middle cerebral aneurysm (1 case with ipsilateral anterior A2 cerebral aneurysm)**	2 (18.2%)
**Patients with left anterior choroidal aneurysm**	1 (9.1%)
**Patients with anterior communicating artery aneurysm**	2 (18.2%)
**Patients with right posterior communicating artery aneurysm**	2 (18.2%)
**Patients with unruptured aneurysms**	5 (45.5%)
**Mean aneurysm size (mm)**	5.7
**Right transradial access**	11 (100%)
**Stent-assisted coiling**	8 (72.7%)
**Puncture-related complications (Hematoma or radial artery occlusion)**	0
**Transradial access related neurological complications (cerebral infarction, hemorrhage, vasospasm or dissection)**	0

Among 11 patients, five patients failed the treatment via the transfemoral artery approach and were switched to the transradial artery approach, and six patients chose the transradial artery approach because of the difficulty of the transfemoral artery approach. All 11 patients had experienced difficulty in navigating the guiding catheter to the right location for embolization through transradial access, instability, or easy herniation of the guiding catheter into the aorta even when the catheter was in place. The endovascular treatment thus failed without intra-aortic looping of the guiding catheter.

**Figure 1:**
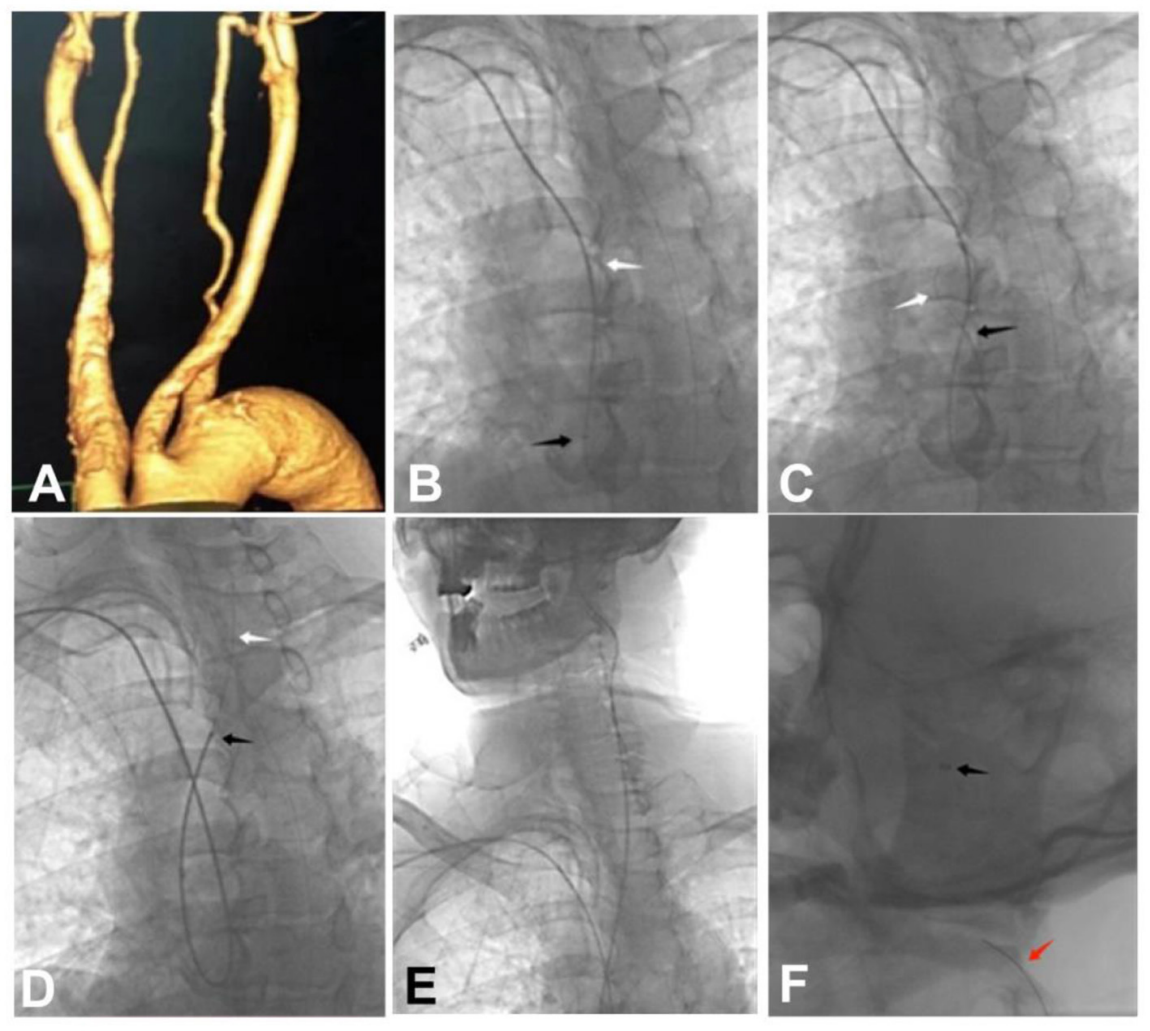
A 59-year-old patient with a left middle cerebral artery aneurysm and a left anterior cerebral artery A2 aneurysm was treated endovascularly through the right transradial access with intra-aortic catheter looping because of the difficulty of embolization via the transfemoral access. **(A)** Three-dimensional reconstruction angiography revealed a type III aortic arch with widened aortic arch. **(B)** A 6F115 cm Navien intermediate catheter was guided by a guide wire to form a loop within the aortic lumen. **(C)** The Navien intermediate catheter was slowly introduced by the guide wire to form a loop within the aorta. **(D)** The guide wire and the Navien intermediate catheter were used to superselect the left common carotid artery. **(E, F)** The tip of the Navien intermediate catheter was used to superselect the internal carotid artery, and after the catheter tip was placed at the C2 segment, a V18 guide wire was sent into the Navien intermediate catheter. Black arrow: the tip of the Navien intermediate catheter; White arrow: the guide wire; Red arrow: the V18 guide wire.

**Figure 2:**
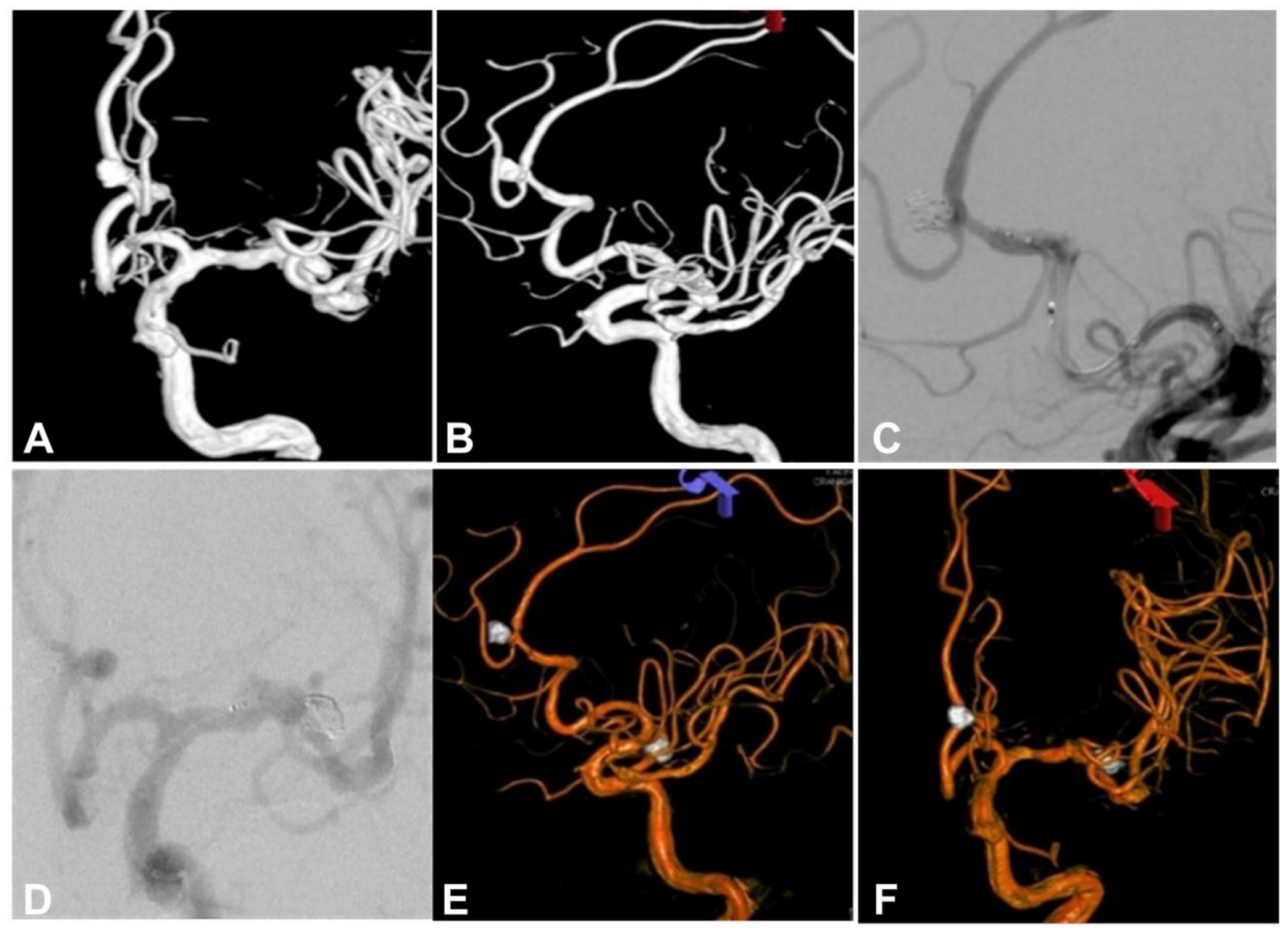
Same patient as in Figure 1. **(A, B)** Three-dimensional reconstruction cerebral angiography showed a left middle cerebral aneurysm and a left anterior cerebral artery A2 aneurysm. **(C)** A LVIS Jr stent was used to assist coil embolization of the left anterior cerebral artery A2 segment aneurysm. **(D)** The left middle cerebral artery aneurysm was embolized with stent-assisted coiling by the use of another LVIS stent. **(E, F)** Three-dimensional reconstruction cerebral angiography after embolization of both aneurysms revealed complete occlusion of both aneurysms.

**Figure 3:**
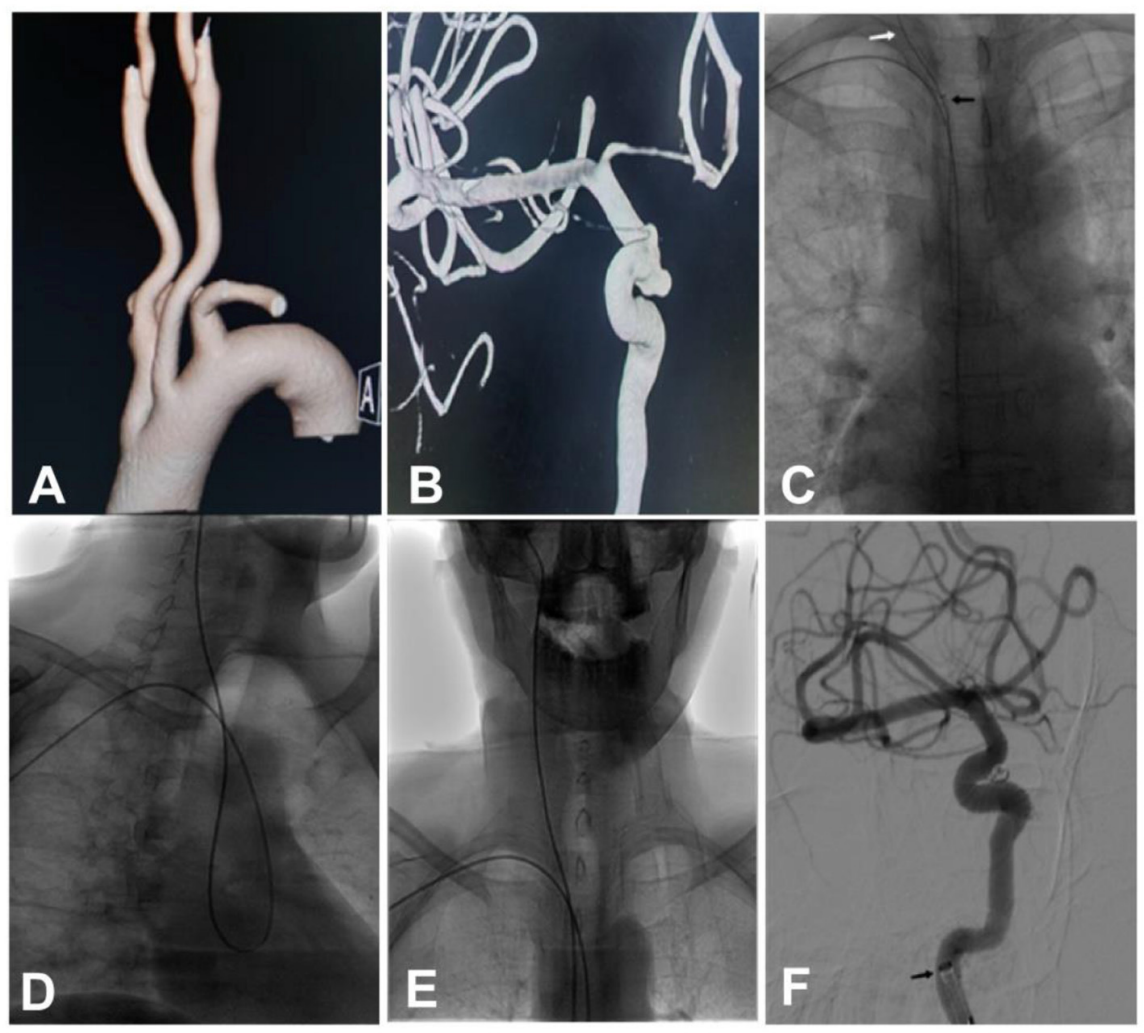
A female patient in their 50s had an intracranial aneurysm of the right internal carotid artery (ICA) which was found during the physical examination. **(A)** A Type III aortic arch was demonstrated on reconstruction cerebral angiography. **(B)** Angiography showed a wide-necked aneurysm in the intracranial segment of the right ICA, with irregular aneurysm morphology. **(C)** A loach guide wire combined with a 6F115-cm Navien catheter was looped within the ascending aorta. **(D, E)** A 6F 115 cm Navien microcatheter was superselected into the right ICA. **(F)** Stent-assisted embolization of the aneurysm was performed. Black arrow: the head of the Navien catheter; white arrow: the loach guide wire.

The right radial artery approach was adopted in all patients, and the success rate of intra-aortic catheter looping was 100%. The embolization of intracranial aneurysms was successfully completed in all patients (Figures 1–3). No instability of the guide catheter was encountered, and the heart rate and blood pressure of all patients were stable during the endovascular operation. No puncture site complications or surgical-related neurological complications occurred after the treatment.

## Discussion

In this study investigating the feasibility and effect of transradial access with intra-aortic catheter looping for the treatment of intracranial aneurysms, it was found that transradial access with intra-aortic catheter looping for embolization of intracranial aneurysms was technically feasible, safe, and efficient as an important supplementary approach to the routine transfemoral access or transradial access without intra-aortic catheter looping.

For cerebrovascular diagnosis and treatment through transradial access, the guiding catheter should first be turned downward through the subclavian or innominate artery to enter the aorta before being turned upward to get into the target vessel. Most cerebral angiography via transradial access only needs to use the reverse curved catheter to select the artery opening through “rotation and lifting” without further superselection, which is relatively simple and can meet the clinical diagnostic needs. However, many requirements need to be met on the guiding catheter during cerebral aneurysm embolization through transradial access: (1) the target vessel should be superselected, and the catheter tip should be placed as close to the aneurysm as possible and (2) it should form stable support on the blood vessel wall so as to provide sufficient support during endovascular embolization of the aneurysm. For patients with a blunt arterial angle formed between the subclavian or innominate artery and the common carotid artery, it is easy to navigate the guiding catheter to the right place to obtain sufficient support for endovascular embolization if the left carotid artery of the bovine arch is superselected through transradial access on the right side (11). However, when the arterial angle formed between the subclavian or innominate artery and the common carotid artery is small, it is relatively difficult to send the guiding catheter to the right place, and it requires a hard guiding catheter to change the local vascular morphology and form a stable support on the vascular wall for endovascular operation (22). For patients with a sharp arterial angle or a long distance between the innominate artery and the left common carotid artery or for patients with severe calcification of the aorta and arterial branches above the arch, it is often difficult to successfully navigate a hard guiding catheter in place and form a stable support, without risking vascular injury (22, 23). In these cases, transradial access with intra-aortic catheter looping would be helpful for successful catheter navigation and stable support for endovascular embolization operation.

It has been reported that the catheter looping technique is not limited by the above anatomical characteristics in endovascular embolization for carotid artery stenting or cerebral angiography (14–18). After the catheter forms a larger loop in the ascending aorta, the top of the catheter loop is supported by the aortic arch, thus forming a strong support for endovascular embolization operation. When the guiding catheter forms a loop within the aorta, the catheter tip is aligned with the direction of blood flow, and it is thus easy to superselect the target arterial branch on the aortic arch. In the literature, an exchange technique is used in the looping of a guiding catheter: a small-diameter catheter was used to form a loop within the aorta before superselecting the target vessel. After that, a harder exchange guide wire was used to guide a harder guiding catheter into a loop before entering the target vessel. If the support of a single guide wire was insufficient, the technique of double guide wires or balloon anchoring was required (14, 17, 19, 21).

In using the aortic looping technique, the guide catheter has no adverse effect on the aortic valve activity. In the literature (14–17, 19), when using this method for stent implantation in the internal carotid artery or common carotid artery, the stent system used was relatively hard, but the guiding catheter was relatively stable, with no report of the catheter herniating into the ventricle. The support required by the microcatheter for intracranial aneurysm embolization is relatively small. Using this method, the guiding catheter can provide sufficient support for intracranial aneurysm embolization. Nonetheless, attention should be paid to the position of the catheter head during operation to avoid dangerous events caused by extreme operation.

In this study, the softer Navien intermediate catheter was used instead of an ordinary harder guiding catheter. With this softer Navien catheter, the use of a loach guide wire only was able to complete the looping process and superselection of the target artery, avoiding the use of the exchange technique and making endovascular operation simpler and less irritating to the blood vessel wall. In addition, the Navien intermediate catheter can be successfully navigated in place for patients with tortuosity of the common and internal carotid arteries. Nonetheless, the Navien intermediate catheter itself is soft and has a weaker axial support force. After the Navien intermediate catheter is sent in place, a V18 guide wire is placed within the Navien intermediate catheter which serves as a guiding catheter (guide wire retention), thus enhancing the support of the intermediate catheter and avoiding folding of the catheter. The lumen of a 6F Navien intermediate catheter is large enough, and the aneurysm embolization operation will not be affected after a V18 guide wire is retained within the intermediate catheter.

When performing intra-aortic catheter looping, a relatively long guiding catheter is required. When patients are ≤170 cm in height, a 115-cm intermediate catheter is sufficient. If the height of the patient is ≤175 cm, a 125-cm intermediate catheter is required. If the height is over 175 cm, the length of a microcatheter may be insufficient to reach the aneurysm, and a puncture of the radial artery or brachial artery at a higher location is required (14, 15). This intra-aortic catheter looping technique is prohibited for patients with aortic valve insufficiency, valvular vegetation, or ascending aortic disease. The use of a hard catheter for looping may cause aortic injury with disastrous consequences.

Some limitations existed in this study, including the retrospective and one-center study design, Chinese patients enrolled only a small cohort of patients, no randomization, or control, which may all affect the generalization of the outcome. Future prospective, randomized, controlled clinical trials with a large sample are necessary to confirm the outcome of this study.

In conclusion, transradial access with intra-aortic catheter looping for embolization of intracranial aneurysms is technically feasible, safe, and efficient and can be used as an important supplementary approach to routine transfemoral access or transradial access. Nonetheless, as a new surgical method, studies with a large sample are needed to confirm the safety and effectiveness of this approach.

## Data availability statement

The raw data supporting the conclusions of this article will be made available by the authors, without undue reservation.

## Ethics statement

The studies involving human participants were reviewed and approved by the Ethics Committee of Henan Provincial People's Hospital. The patients/participants provided their written informed consent to participate in this study.

## Author contributions

Study design: G-QX and T-XL. Data collection: G-QX, Y-YB, D-YC, B-WY, T-YZ, and J-YX. Revision: B-LG. Writing of the original version: G-QX. Data analysis: G-QX and B-LG. Validation: all authors. All authors contributed to the article and approved the submitted version.
